# Histidine-tryptophan-ketoglutarate solution decreases mortality and morbidity in high-risk patients with severe pulmonary arterial hypertension associated with complex congenital heart disease: an 11-year experience from a single institution

**DOI:** 10.1590/1414-431X20165208

**Published:** 2016-05-13

**Authors:** X.W. Li, Y.Z. Lin, H. Lin, J.B. Huang, X.M. Tang, X.M. Long, W.J. Lu, Z.K. Wen, J. Liang, D.Y. Li, X.F. Zhao

**Affiliations:** 1 Department of Cardiothoracic Surgery, People's Hospital of Guangxi Zhuang Autonomous Region, Nanning, Guangxi, China; 2 Department of Cardiothoracic Surgery, Ruikang Hospital, Affiliated to Guangxi University of Chinese Medicine, Nanning, Guangxi, China; 3 Pediatric Center of Cardiac Surgery, Cardiovascular Institute and Fuwai Hospital, Chinese Academy of Medical Sciences and Peking Union Medical College, Beijing, China

**Keywords:** Histidine-tryptophan-ketoglutarate, Myocardial protection, Cardiac surgery

## Abstract

Cardioplegic reperfusion during a long term ischemic period interrupts cardiac surgery and also increases cellular edema due to repeated solution administration. We reviewed the clinical experiences on myocardial protection of a single perfusion with histidine-tryptophan-ketoglutarate (HTK) for high-risk patients with severe pulmonary arterial hypertension associated with complex congenital heart disease. This retrospective study included 101 high-risk patients undergoing arterial switch operation between March 2001 and July 2012. We divided the cohort into two groups: HTK group, myocardial protection was carried out with one single perfusion with HTK solution; and St group, myocardial protection with conventional St. Thomas' crystalloid cardioplegic solution. The duration of cardiopulmonary bypass did not differ between the two groups. The mortality, morbidity, ICU stay, post-operative hospitalization time, and number of transfusions in HTK group were lower than those in St group (P<0.05). Univariate and multivariate analysis showed that HTK is a statistically significant independent predictor of decreased early mortality and morbidity (P<0.05). In conclusion, HTK solution seems to be an effective and safe alternative to St. Thomas' solution for cardioplegic reperfusion in high-risk patients with complex congenital heart disease.

## Introduction

Myocardial protection is associated with increased mortality and morbidity, and is always an important issue to consider in cardiac surgery, especially in long ischemic periods followed by reperfusion. However, the optimal myocardial protection strategy for high-risk patients with severe pulmonary arterial hypertension associated with complex congenital heart disease undergoing cardiac surgery remains controversial (1,2). In recent years, an increasing number of patients with complex congenital heart disease underwent cardiac surgeries under cardiopulmonary bypass (CPB) (2,3). The improvement of myocardial protection for these patients is a key issue that needs consideration (4). We hypothesized that a single dose of histidine-tryptophan-ketoglutarate (HTK) could offer superior myocardial protection than cold crystalloid cardioplegic solution in these high-risk patients. The purpose of this study was to compare myocardial protection of HTK and St. Thomas' crystalloid cardioplegic solution in high-risk patients with severe pulmonary arterial hypertension associated with complex congenital heart disease.

## Material and Methods

### Patients

Mean pulmonary artery pressure >50 mmHg or systolic pulmonary/systemic artery pressure ratio >0.8 from cardiac catheterization data of patients under general anesthesia were used to define severe pulmonary arterial hypertension. A total of 101 high-risk patients (aortic cross-clamp time of 90 min) older than 6 months with dextro-transposition of the great arteries (d-TGA) and nonrestrictive ventricular septal defect, or Taussig-Bing anomaly and severe pulmonary arterial hypertension undergoing arterial switch operation (two ventricles repair) at Fuwai Hospital from March 2001 to July 2012 were included in the study. Patients were divided into two groups: HTK group (myocardial protection was carried out with one single perfusion of HTK solution) and St group (control group, myocardial protection with conventional St. Thomas' cold potassium crystalloid cardioplegic solution) (Table 1). Criteria for patient selection included: patients older than 6 months diagnosed as d-TGA and nonrestrictive ventricular septal defect or Taussig-Bing anomaly and severe pulmonary arterial hypertension. Criteria for exclusion were: patients younger than 6 months, patients with mean pulmonary arterial pressure <50 mmHg or systolic pulmonary/systemic artery pressure ratio <0.8, and patients with trisomy 21. The diagnosis was based on echocardiographic and cardiac angiographic findings, and confirmed during operation. The surgical team was the same in both groups.

**Table:**
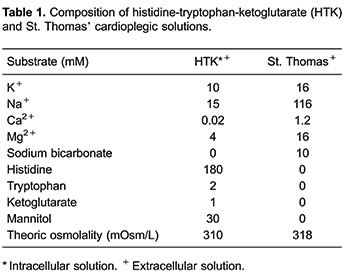

Early mortality was defined as death before hospital discharge or within 30 days of arterial switch operation. The study protocol was approved by the Medical Ethics Committee of Fuwai Cardiovascular Disease Hospital, which provided the approval to waive the need for patient consent for publishing retrospective follow-up data about these patients. The records of all patients were reviewed retrospectively.

### Anesthesia management

Anesthesia was induced with intravenous 1.5 mg/kg ketamine, 30 µg/kg fentanyl, and 0.15 mg/kg pancuronium, and was maintained with 0.3 µg·kg^-1^·min^-1^ fentanyl during surgery. Hemodynamic monitoring was carried out *via* arterial line in the radial artery or femoral artery; a central venous pressure catheter was installed *via* subclavian vein; and a urinary catheter was installed.

### Cardiopulmonary bypass

Cardiopulmonary bypass was set up by an arterial cannula in the aortic root, and venous drainage was obtained from superior and inferior vena cava. Cardiopulmonary bypass was performed using a hollow fiber oxygenator, Dideco 901 or 902 (Sorin, Italy), and a roller pump (Jostra, Germany) with nonpulsatile flow for both groups. Tubing pack with crystalloid cardioplegia delivery system (Perfect, China), and an arterial filter (Xi Jing, China) were used. A hemo-concentrator (Gambro, Germany) was placed with the inlet connected to the arterial line and outlet to the venous line for both groups. Activated clotting time was measured by a Hemochron (Gambro) and was maintained above 400 s during cardiopulmonary bypass. After cardiopulmonary bypass, heparin was neutralized with 4 mg/kg protamine chloride.

### Infusion of cardioplegia

HTK solution (4–8°C) was perfused at an initial perfusion pressure of 80–100 mmHg and was maintained at 40–60 mmHg during myocardium arrest, and was infused over 5-7 min. All cases were perfused with a single dose of 40-50 mL/kg. St. Thomas' solution (4–8°C) was perfused antegrade every 30 minutes at an initial dose of 20 mL/kg and maintenance at a dose of 10 mL/kg, with perfusion pressure of 100–120 mmHg.

### Surgical technique

Median sternotomy and hypothermic cardiopulmonary bypass with ultrafiltration technique were routinely used. Surgery was performed on cardiopulmonary bypass at low flow (50 mL·kg^-1^·min^-1^) with a rectal temperature of 18–22°C. Ventricular and atrial septal defect and patent ductus arteriosus were completely closed, and there were no fenestrations. The aorta was transected and the aortic root fully dissected. A large aortic button containing the coronary orifice was excised. The pulmonary trunk was transected, pulmonary branches dissected, and the Lecompte maneuver performed. The coronary artery button was reimplanted in the appropriate site of the neo-aorta. Pulmonary artery reconstruction was done with a fresh autologous pantaloon shaped pericardial patch. The associated anomalies were corrected simultaneously.

### Follow-up

All survivors discharged from the hospital were followed up to the end date of the study. All patients at the outpatient department were examined with electrocardiogram, X-ray chest film and echocardiogram, once every 3 months. At the last follow-up, the patients were contacted by telephone or interviewed directly at the outpatient department.

### Statistical analysis

All analyses were performed using SPSS version 18.0 software (SPSS Inc., USA). Continuous variables are reported as means±SE and compared by a two-tailed Student's *t-*test. Survival rates were estimated using the Kaplan-Meier method. Comparison of multiple mean values was carried out by ANOVA. Discrete variables are reported as percentages and compared by the Fisher's exact test or the Pearson's χ^2^ test as required. The relationships with perioperative risk factors were assessed by means of contingency table methods and logistic regression analysis. To explore the simultaneous effects of perioperative characteristics on early death, variables that were significant at the 0.1 level in univariate analysis were included in a multivariate logistic regression model. P values less than 0.05 were considered to be statistically significant.

## Results

Table 2 reports the demographic and operative data. Preoperative cardiac catheterization data were available for all patients. Preoperative pulmonary artery pressure and pulmonary vascular resistance were measured by the conventional cardiac catheterization protocol and Fick method.

**Table:**
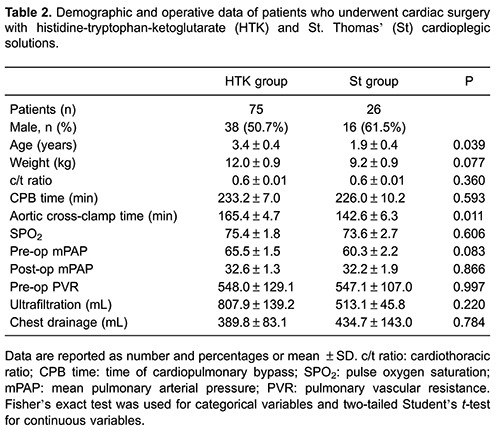

There were 6 operative deaths. Mortality in the HTK group was significantly lower than that in the St group (2.7 versus 15.4%, P=0.037), and multiple organ failure in HTK group was significantly lower than that in St group (P=0.040*)*. The incidence of morbidity in the HTK group was significantly lower than that in the St group (24 versus 65.4%, P=0.000; Table 3).

**Table:**
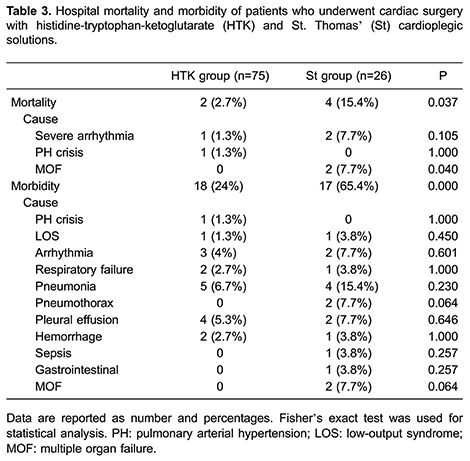

ICU stays for the HTK group were significantly shorter than those of the St group (10.41±1.36 *vs* 20.46±3.63 days, P=0.002). The mean volume of transfusion for patients was significantly less in the HTK group (760.67±83.28 *vs* 1520.00±174.83 mL, P=0.000; Table 4). Table 5 shows the change in serum sodium in the HTK group.

**Table:**
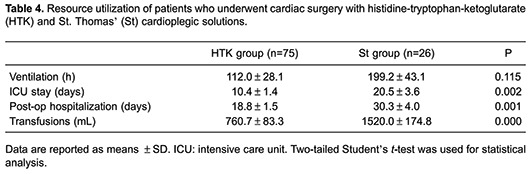

**Table:**
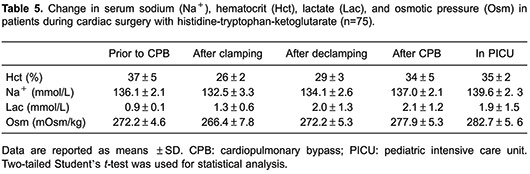

After reperfusion, rates of spontaneous defibrillation of the HTK group were significantly higher than those of the St group (92.1 *vs* 73.1%, P=0.036).

Complete follow-up was achieved for 90.5% (86/95) of cases. The mean duration of follow-up was 56.1±28.6 months; 2 late deaths occurred due to sudden death. Survival rate was 97.7% (84/86). The latest follow-up data showed that 2.4% of survivors were in the NYHA (New York Heart Association) class II and 97.6% in class I.

Both univariate and multivariate analysis showed that HTK was associated with decreased early mortality (OR= 0.151, P=0.035) and decreased morbidity (OR=0.167, P=0.000).

## Discussion

HTK solution, which was introduced by Bretschneider in 1975, belongs to the cardioplegic solutions based on intracellular electrolytes (5). It is also used as a multiorgan preservation solution.

In 1965, Issellhard et al. confirmed that the depletion of high energy phosphate accelerates with the increase of K^+^ concentration (6). When the extracellular K^+^ concentration is above 50 mmol/L, and the ventricular wall tension increases, Ca^2+^ accumulates in the myocardial cells. The explanation for this is that the extracellular K^+^ concentration increases and the membrane potential decreases, resulting in the opening of voltage-gated calcium channel (7,8). del Nido et al. (9) also confirmed that high potassium reperfusion can lead to intracellular calcium overload because of abnormal calcium. Jellinek (8) also reported that high potassium solution resulted in myocardial and coronary artery endothelial damage. Bretschneider et al. (5) have shown that the concentration of potassium of 8–12 mmol/L is enough to cause heart arrest. Therefore, the KCl concentration of 10 mM in HTK solution efficiently causes heart arrest with the minimum damage to cells (9,10). The concentration of Na^+^ in HTK solution is 15 mM, which is similar to that of the cell. Na^+^ plays an important role in the formation of cardiac action potential, and fast action potential causes the rapid depolarization of the myocardium. Low sodium can reduce concentration differences of Na^+^ across the membrane, not only reducing H^+^-Na^+^ exchange as a result of high potassium, but also alleviating Na^+^ influx caused by the electrochemical gradient of Na^+^. Therefore, the action potential cannot be generated, and the heart arrests in a low potassium concentration of the diastolic phase. These two mechanisms can reduce the intracellular Na^+^ concentration, so HTK with low Na^+^ can inhibit Na^+^/Ca^2+^ exchange, reducing the intracellular calcium overload during ischemia and reperfusion. In addition, less Na^+^ influx also reduces cellular edema during ischemia. The HTK solution, which has no calcium, is conducive to reducing intracellular calcium influx, intracellular calcium overload and myocardial injury resulting from high calcium ion concentration (11,12).

Histidine present in the HTK solution can enhance the efficiency of anaerobic glycolysis, while tryptophan stabilizes cell membrane, and the addition of mannitol decreases cellular edema. Histidine, a protein buffer, might be superior to bicarbonate in stabilizing intracellular pH and facilitating preservation of myocardial adenosine triphosphate storage. Also, histidine improves post-arrest contractile function and minimizes myocardial necrosis (11,13). HTK solution also preserves the coronary artery endothelium and improves functional cardiac recovery (12,14). A clinical study indicated that the use of the HTK solution yielded a lower incidence of arrhythmias, shorter length-of-stays in the intensive care unit, and less post-operative inotropic support (15).

Studies with sufficient number of patients and adequate postoperative measurements to detect significant adverse effects of cardioplegia for high-risk patients with complex congenital heart disease are lacking (1,16,17). Data on the use of the HTK solution in these patients are rare (18,19).

A study from our institution compared the myocardial protection of the HTK solution and conventional St. Thomas' crystalloid cardioplegia during the long-term ischemic period (cross-clamping time >90 min) in complex pediatric heart surgery without pulmonary arterial hypertension. The results demonstrated that mortality and the level of creatine kinase in the HTK solution group was significantly lower than those in St. Thomas' crystalloid cardioplegia group (P<0.05). Patients with severe pulmonary arterial hypertension associated with congenital heart disease are difficult patients in clinical practice. Postoperative pulmonary arterial hypertension is, in fact, a major determinant of perioperative morbidity and mortality (20).

A single dose perfusion (40–50 mL/kg) with the HTK solution can cause hemodilution and hyponatremia. Special attention to the lower Na^+^ concentration is required. However, hyponatremia has few negative effects on the clinical outcome (21). "Over treatment" of such low sodium content – especially in pediatrics – must be avoided, especially if the osmolarity is within a physiologic range, to prevent severe neurologic complications (22).

St. Thomas' cardioplegia is still in use in our hospital, though we have been using blood cardioplegia more often. Cold crystalloid cardioplegia is one of the earliest and most widely used cardioplegia. It can cause heart arrest in the diastolic phase due to the high concentration of potassium. St. Thomas' cardioplegia is the most common crystalloid cardioplegia, which has a good myocardial protection effect, it is of simple administration and widely used, and is especially suitable for open heart surgery with short intraoperative time. However, crystalloid cardioplegia can lead to myocardial foci of necrosis, injury to coronary artery endothelium, and release of inflammatory mediators and aggregation of leukocyte, therefore it is being gradually replaced by other new cardioplegia.

Blood cardioplegia provides oxygen and matrix for the myocardium and reduces damage to myocardial cells. It mimics the physiological matrix and trace elements, it is easy to prepare, and inexpensive. Disadvantages of blood cardioplegia include imperfect cardiac arrest and dim operation field. A large number of red blood cells in blood cardioplegia increases blood viscosity in low temperatures, which affects the effective and uniform perfusion of the myocardium. In addition, activated white blood cells in blood cardioplegia are one of the main factors that cause systemic inflammatory response syndrome induced by cardiopulmonary bypass.

Our study showed that the HTK solution decreased mortality, morbidity, ICU stay, post-operative hospitalization, and number of transfusions in high-risk patients with complex congenital heart disease. A prospective randomized trial should be carried out to certify the benefits of HTK solution compared to St. Thomas' crystalloid cardioplegia for high-risk cardiac patients.
